# Construction of a Feline Amniotic Membrane Scaffold Loaded with Limbal Stem Cell Grafts

**DOI:** 10.3390/vetsci13090853

**Published:** 2026-08-23

**Authors:** Can Huang, Xiaoyan Hu, Yunying Peng, Xueling Piao, Xuanrui Tao, Shihao Yang, Heng Yang, Shicheng Bi, Dezhi Zhang, Lijing Cao, Bin Wu, Ling Gan, Huihao Xu

**Affiliations:** 1 College of Veterinary Medicine, Southwest University, Chongqing 402460, China; 13594612901@163.com (C.H.); 17702348933@163.com (X.H.); pengyunying429@163.com (Y.P.);; 2 Rongchang District Vocational Education Center, Chongqing 402460, China; 3 Chongqing Medical and Pharmaceutical College, Chongqing 401331, China; 4 Molecular Engineering and Vaccine Innovation Center for Luminescent Animals, Southwest University, Chongqing 400700, China

**Keywords:** epidermal growth factor, feline, corneal limbal stem cells, lyophilized amniotic membrane, transplant material

## Abstract

Amniotic membranes are extensively used in feline corneal repair; however, challenges such as rapid degradation and difficulties with preservation across species limit their clinical use. The aim of this study was to develop a feline amniotic membrane-based graft with stem cell activity and provide experimental evidence for its potential use as a corneal epithelial transplant material in cats. FLSCs were first treated with different concentrations of epidermal growth factor (EGF), and 0.5 mg/L EGF was identified as the optimal concentration for promoting cellular activity. Under this condition, FLSCs exhibited enhanced proliferation and wound closure ability, with complete scratch closure observed approximately 24 h earlier than that in the control group. Growth curve analysis showed that FLSCs maintained stable proliferation characteristics, with a calculated population doubling time of approximately 25.2 h. Subsequently, FLSCs were seeded onto the lyophilized feline amniotic membrane scaffold. Light microscopy, scanning electron microscopy, and transmission electron microscopy demonstrated that FLSCs adhered to and proliferated on the scaffold, forming cell–matrix interaction structures, including hemidesmosome-like junctions. Immunofluorescence staining confirmed the expression of stem cell-associated markers ABCG2 and p63 after scaffold culture. These findings demonstrate the successful fabrication of a feline FLSC-loaded amniotic membrane construct in which FLSCs retained the expression of the examined limbal/stem cell-associated markers after scaffold culture. The successfully constructed FLSC-loaded feline amniotic membrane graft demonstrated considerable potential as a novel bioengineered tissue substitute for corneal surface transplantation in clinical applications.

## Simple Summary

Corneal surface injuries are common in cats and can be difficult to treat when the damaged cornea does not heal properly. The amniotic membrane is widely used to support corneal repair, but its preservation and compatibility with feline tissues remain important challenges. In this study, we developed a feline amniotic membrane scaffold loaded with immortalized feline limbal stem cells (FLSCs). We first identified an appropriate concentration of epidermal growth factor to support FLSC growth and found that 0.5 mg/L EGF enhanced cell proliferation and wound closure. The FLSCs were then seeded onto a lyophilized feline amniotic membrane scaffold. Microscopic examination showed that the cells attached to and grew on the scaffold, while immunofluorescence staining confirmed the expression of the limbal/stem cell-associated markers ABCG2 and p63 after scaffold culture. These results indicate that the lyophilized feline amniotic membrane can support FLSC attachment and growth while maintaining the expression of the examined stem cell-associated markers. This construct may provide a useful basis for developing a cell-loaded biological scaffold for future treatment of feline corneal surface diseases.

## 1. Background

Feline corneal disease is a frequently encountered ophthalmic condition in veterinary practice. It can cause vision impairment and, in advanced cases, blindness. Severe lesions may compromise ocular integrity and, in rare circumstances, threaten overall health [1]. Superficial corneal diseases in cats are managed by conservative medical therapy and surgical interventions. Most complex corneal diseases cannot be cured solely by medication and frequently necessitate corneal surgery and patch transplantation to achieve therapeutic effects [2]. Amniotic membranes have been used for feline corneal repair owing to their avascular nature, ability to promote epithelialization, and anti-inflammatory, antifibrotic, and anti-protease properties [3,4,5,6]. However, their broader clinical application has been limited by the potential immunogenicity of xenogeneic amniotic tissues, their susceptibility to rapid biodegradation, and the technical challenges associated with long-term preservation [7,8]. Limbal stem cells (LSCs) are a key source of epithelial repair and have attracted considerable attention in the field of ophthalmology for many years owing to their applications in corneal diseases, cell culture, ocular surface transplantation, and drug development [9,10]. Amniotic membrane-based limbal stem cell culture systems and ocular surface reconstruction approaches have been investigated in humans, rabbits, dogs, and several other species [11,12,13]. However, studies focusing on feline-derived amniotic membrane scaffolds remain very limited. With the increasing clinical importance of companion animals, particularly cats, the development of species-specific biomaterial scaffolds may improve biological compatibility and translational applicability in veterinary ophthalmology. In addition, freeze-drying may enhance the storage stability and clinical accessibility of amniotic membrane materials, thereby facilitating the future development of off-the-shelf tissue-engineered ocular surface grafts [14]. However, whether a lyophilized feline amniotic membrane can effectively support FLSC growth and maintain stem cell characteristics remains unclear. Therefore, the present study was designed based on the hypothesis that a species-specific lyophilized feline amniotic membrane could provide a suitable extracellular matrix environment for FLSC adhesion, proliferation, and maintenance of stem cell characteristics. To test this hypothesis, we first evaluated the biological effects of EGF on FLSC proliferation and migration, then constructed an FLSC-loaded amniotic membrane scaffold, and finally characterized cellular attachment, ultrastructural interactions, and stem cell marker expression to preliminarily assess its feasibility as a tissue-engineered corneal graft.

## 2. Methods

**Figure 1:**
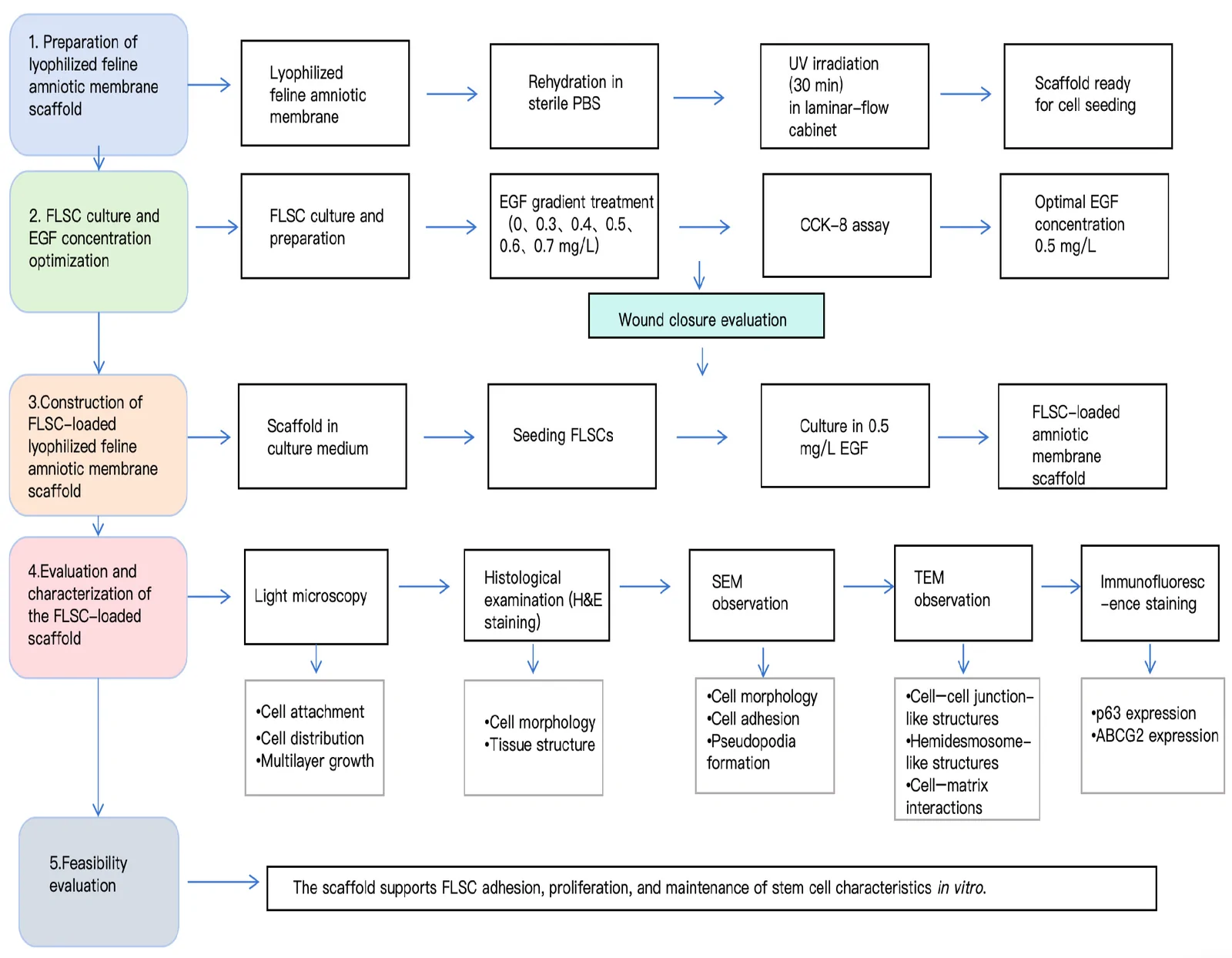
Schematic illustration of the construction and characterization workflow of the FLSC-loaded lyophilized feline amniotic membrane scaffold.

The overall experimental workflow of scaffold preparation, FLSC culture, scaffold construction, and subsequent characterization procedures is summarized in Figure 1. The experimental design was divided into three sequential stages: (1) optimization of FLSC biological activity by EGF stimulation; (2) construction of the FLSC-loaded lyophilized feline amniotic membrane scaffold; and (3) evaluation of scaffold compatibility through morphological observation, ultrastructural analysis, and stem cell marker detection.

### 2.1. Key Reagents and Instruments

Immortalized feline limbal stem cells (FLSC-SV40LT), previously established and characterized by our research group, were used in this study. Briefly, primary feline limbal stem cells were obtained from the limbal tissues of three healthy Chinese domestic cats. The limbal tissues were collected in vivo under general anesthesia following OCT-based localization of the limbal stem cell region and surgical excision. The primary feline limbal stem cells were subsequently immortalized using SV40 large T antigen (SV40LT). The established FLSC-SV40LT cell line was previously characterized and shown to exhibit stable proliferative capacity after immortalization and to retain limbal stem cell-associated characteristics, including the expression of p63 and ABCG2 [15]. The establishment and characterization of the FLSC-SV40LT cell line were conducted in accordance with the standards of the Southwest University Institutional Animal Care and Use Committee (IACUC), under approval number IACUC-20220930-02 [15]. No additional tissue collection from live animals was performed in the present study.

Before scaffold seeding, the FLSC-SV40LT cells had been previously characterized for the expression of limbal stem cell-associated markers, including p63 and ABCG2 [15]. In the present study, cells between passages 7 and 10 (P7–P10) were used for all experiments. The cell line was maintained and expanded under standard culture conditions before scaffold construction experiments. Dedicated mycoplasma testing and formal cell-line authentication were not performed in the present study; therefore, these quality-control parameters could not be independently verified in the current study. The identity and baseline characteristics of the FLSC-SV40LT line were based on the previously published establishment and characterization study [15].

The FLSCs were maintained and expanded under standard culture conditions before scaffold construction experiments. The lyophilized feline amniotic membrane scaffold used in this study was co-developed by our laboratory in collaboration with Shanghai Kangterunmu Medical Technology Co., Ltd., Shanghai, China The scaffold was derived from feline amniotic membranes collected after natural delivery and was processed by the manufacturer into individually packaged lyophilized biological scaffolds. The manufacturer produces the lyophilized amniotic membrane scaffolds from feline amniotic membranes obtained from different donors and batches. In the present study, one representative batch of scaffold material was used for preliminary in vitro evaluation. Before use, the lyophilized amniotic membrane was rehydrated in sterile PBS and handled under aseptic conditions for subsequent cell seeding and characterization experiments. Recombinant human epidermal growth factor (hEGF, 300 mg/L; 30,000 IU/3 mL/vial; Guilin Huanuowei Gene Pharmaceutical Co., Ltd., Guilin, China) was used as a biological supplement to regulate FLSC proliferation and migration.

Cell proliferation was evaluated using a CCK-8 assay kit, Beyotime Biotech Inc., Shanghai, China. Stem cell characteristics were assessed by immunofluorescence staining using antibodies against ABCG2 and p63. Cellular morphology, scaffold structure, and cell–scaffold interactions were examined using light microscopy, scanning electron microscopy, and transmission electron microscopy.

### 2.2. Cell Culture

FLSCs were routinely maintained in T25 cell culture flasks (25 cm^2^ growth area, Corning Inc., Corning, NY, USA) containing complete Dulbecco’s Modified Eagle’s Medium (DMEM) supplemented with 10% fetal bovine serum, 100 U/mL penicillin, and 100 U/mL streptomycin at 37 °C and 5% CO_2_ in a humidified incubator. When cells reached 70–80% confluence, cells were digested with 0.25% trypsin for passage or frozen in liquid nitrogen using serum-free cell cryopreservation solution following digestion. All experiments were conducted using cells in the logarithmic growth phase.

### 2.3. CCK-8 Detection of FLSC Proliferation Under Different Concentrations of EGF

FLSCs in the logarithmic growth phase were digested and resuspended to obtain single-cell suspensions. Cells were seeded into a 96-well plate pre-coated with phosphate-buffered saline (PBS), with the wells allocated as blanks, experimental groups (2 × 10^3^ cells per well), and controls. After incubation at 37 °C under 5% CO_2_ for 24 h, the culture medium was removed, and the wells were gently washed once with PBS. Subsequently, 100 μL of DMEM containing EGF at final concentrations of 0, 0.3, 0.4, 0.5, 0.6, and 0.7 mg/L was added to each well. Recombinant human EGF was used. Wells treated with 0 mg/L EGF served as the control group, and the remaining concentrations constituted the experimental groups. The blank wells contained an equal volume of DMEM without cells. Each experiment was independently repeated three times, and each group contained ten technical replicates within each experiment. After adding the CCK-8 solution to each group, the plates were incubated at 37 °C for 4 h, and the absorbance at 450 nm (*D*_450_) was measured using a microplate reader. Following measurement, the solution in each well was discarded, and freshly prepared working solutions mixed with the CCK-8 culture medium were added. The plates were then incubated for another 4 h, and the *D*_450_ values were measured at 4, 8, and 12 h. Cell proliferation was evaluated under different EGF concentrations based on the *D*_450_ values. The calculation formula was as follows: Cell viability = (*D*_450, test_ − *D*_450, blank_)/(*D*_450, control_ − *D*_450, blank_) × 100.

### 2.4. Preparation of the Growth Curve of FLSCs Under 0.5 mg/L EGF Treatment

FLSCs with good growth status were collected, washed, digested, and counted. Cells were evenly seeded into two 24-well plates at a density of 1 × 10^4^ cells per well. For the control plate, 2 mL of complete culture medium was added to each well. For the EGF-treated plate, 2 mL of freshly prepared complete culture medium containing EGF at a mass concentration of 0.5 mg/L was added to each well. Cells were cultured in a humidified incubator at 37 °C with 5% CO_2_, and the medium was changed every alternate day. During each medium replacement, freshly prepared medium containing 0.5 mg/L EGF was added to maintain continuous EGF stimulation throughout the experiment. Cells were digested and counted at 12 time points: 0, 24, 48, 72, 96, 120, 144, 168, 192, 216, 240, and 264 h. Three replicates were counted at each time point, and the experiment was repeated four times.

### 2.5. Scratch Assay to Evaluate FLSC Growth and Wound Closure

FLSCs in the logarithmic growth phase were uniformly seeded at 1 × 10^5^ cells per well and cultured in a constant-temperature cell culture incubator at 37 °C and 5% CO_2_ for 24 h. When cells had almost completely covered the bottom of the well, a vertical scratch was created using the same 1000 μL pipette tip under consistent operating conditions. Detached cells were removed by washing three times with PBS. The initial scratch areas were visually examined to ensure comparable scratch widths before further culture. Complete culture medium and freshly mixed complete culture medium containing an EGF mass concentration of 0.5 mg/L were added separately, and the culture was continued in the incubator. The growth and wound closure of FLSCs in the scratch area were observed under an inverted phase-contrast microscope at eight time points (0, 4, 8, 12, 24, 36, 48, and 60 h). Representative images were captured from randomly selected fields of view at each time point. The wound closure process was evaluated based on the morphological changes in the scratch gap, including the reduction in scratch width and the degree of gap closure.

### 2.6. Seeding FLSCs onto a Lyophilized Feline Amniotic Membrane Scaffold and Observation Under a Light Microscope

**Figure 2:**
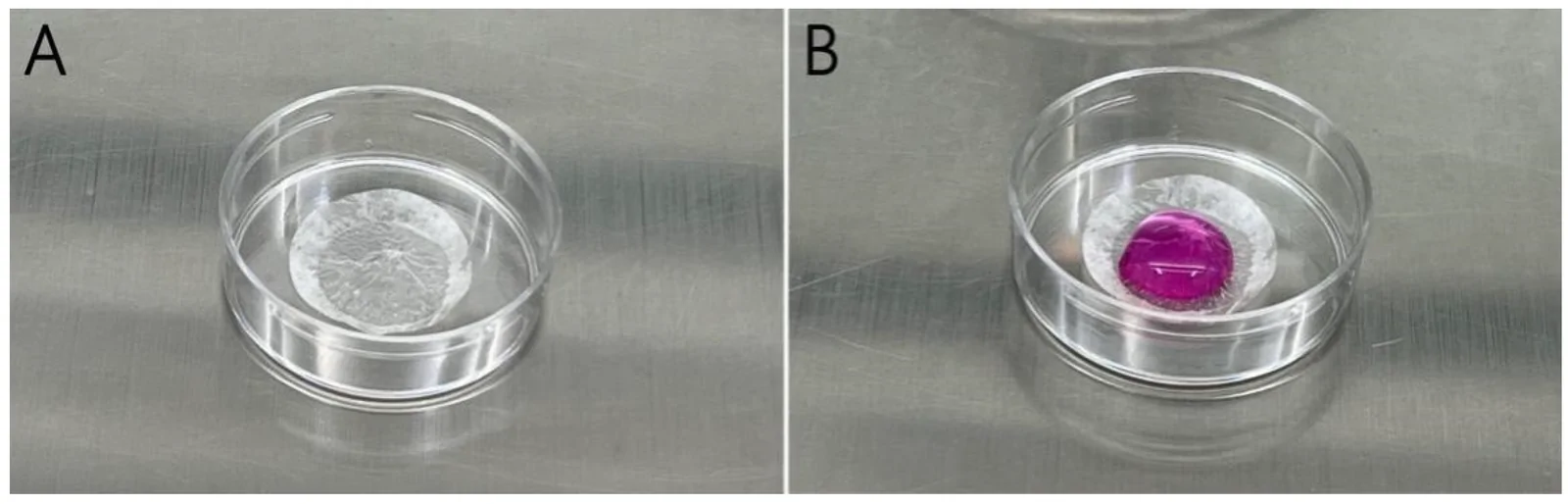
Process of inoculating FLSCs onto a lyophilized feline amniotic membrane scaffold. Panel (**A**) shows the amniotic membrane attached to cell slides, and panel (**B**) shows FLSCs inoculated onto the amniotic membrane.

A sterile cell slide with a diameter of 15 mm was placed at the bottom of a 35 mm Petri dish in advance, and its surface was wetted with PBS. The vacuum-packaged lyophilized feline amniotic membrane scaffold was removed, and the nitrocellulose membrane was opened. The amniotic membrane scaffold attached to the nitrocellulose membrane was placed on the slide with the substrate surface facing downward and the upper epithelial surface facing upward (Figure 2A). The scaffold area was slightly larger than the slide, and the widened part was adjusted to tilt slightly upward to form an amniotic membrane “container.” The attached part was flattened and irradiated with ultraviolet light for 30 min in a laminar-flow cabinet. Subsequently, it was transferred to a 37 °C incubator with 5% CO_2_ and dried for approximately 4 h before its removal for later use. FLSCs in the logarithmic growth phase were routinely digested, and the cell density was adjusted to 1 × 10^5^ cells/mL using EGF-free complete DMEM. The cell suspension was seeded onto the amniotic membrane scaffold (Figure 2B). The Petri dishes were carefully placed in a humidified incubator at 37 °C with 5% CO_2_ for 2–4 h for static culture. After cells settled on the surface of the amniotic membrane scaffold and began to grow, 2 mL of culture medium was added, and the medium was changed every other day. After 3 days of culturing, the culture medium was gently injected from the edge of the amniotic membrane scaffold into the gap between the scaffold and the slide using a pipette, thereby suspending the scaffold at the air–liquid interface and enabling FLSCs to grow on both sides. Cells were cultured for 3–5 days. The culturing process was terminated when cells exhibited multilayer growth and signs of detachment, and the samples were fixed for histological examination.

### 2.7. Histological Observation of FLSCs Seeded on a Lyophilized Feline Amniotic Membrane Scaffold

Tissue sections were prepared using single-sided, cultured, lyophilized feline amniotic membrane scaffolds loaded with FLSCs and fixed in a 10% formaldehyde solution. The fixed amniotic tissue was removed and gently washed under running water for 24 h. The samples were subsequently dehydrated through a graded ethanol series consisting of 70%, 80%, 90%, and 95% ethanol (25 min each), followed by two changes of 95% ethanol and two changes of 100% ethanol (20 min each). The amniotic tissue was immersed in an ethanol–xylene solution until it became completely transparent and was then immersed in a paraffin–xylene solution. The wax-infiltrated amniotic tissue was subsequently placed at the bottom of the embedding mold and allowed to stand until it solidified. The paraffin block was trimmed and sectioned to a thickness of approximately 5 μm. The slices were flattened in a 42 °C constant-temperature water bath, placed on clean glass slides coated with protein glycerol, and allowed to dry. The sections were dewaxed in xylene I and II, followed by ethanol treatment to remove the xylene, washing, and repeated rinsing with distilled water. Finally, the sections were stained with hematoxylin and 0.5% eosin, dehydrated with anhydrous ethanol, cleared in xylene I and II, and sealed with neutral gum. The prepared sections were observed under a biological microscope, and images were collected.

### 2.8. Scanning Electron Microscopy Observation of FLSCs Seeded on a Lyophilized Feline Amniotic Membrane Scaffold

Sterile cell slides with a diameter of 15 mm were placed in a 24-well plate and wetted with PBS, and the excess PBS was aspirated. Circular sections of lyophilized feline amniotic membrane scaffolds with a diameter of 7.0 mm were obtained from the vacuum-packaged membranes using a corneal trephine and attached to the cell slides. After ultraviolet irradiation for 30 min in a laminar-flow cabinet, the samples were transferred to an incubator at 37 °C and dried for approximately 4 h. When the amniotic membrane scaffold was completely attached to the cell slide and could not be easily detached, it was removed for further use. FLSCs in the logarithmic growth phase were routinely digested, and the cell density was adjusted to 1 × 10^5^ cells/mL using complete culture medium containing 0.5 mg/L EGF or without EGF. Cell suspensions were carefully dropped onto an amniotic membrane scaffold to ensure that the culture medium did not overflow through the edge of the membrane. The samples were allowed to stand in a humidified incubator at 37 °C with 5% CO_2_ for 20 min, after which 1 mL of the culture medium was added for continued culturing. The medium was changed every other day, and three wells were set up for each culture condition. After 5–7 days of culturing, when cells exhibited multilayer growth and signs of detachment, the culture process was terminated, and the samples were fixed with 3% glutaraldehyde for scanning electron microscopy.

### 2.9. Transmission Electron Microscopy Observation of FLSCs Seeded on a Lyophilized Feline Amniotic Membrane Scaffold

Cell culture chambers with a diameter of 6.5 mm were placed in a 24-well plate and wetted with PBS, and excess PBS was aspirated. Circular sections of lyophilized feline amniotic membrane scaffolds with a diameter of 5.0 mm were prepared from vacuum-packaged membranes using a corneal trephine and attached to the polycarbonate membrane inside the chamber. The samples were irradiated with ultraviolet light for 30 min in a laminar-flow cabinet and then transferred to an incubator at 37 °C to dry for approximately 4 h. When the amniotic membrane scaffold was completely attached to the polycarbonate membrane and could not be easily detached, it was removed for further use.

FLSCs in the logarithmic growth phase were routinely digested, and the cell density was adjusted to 2 × 10^3^ cells/mL using complete culture medium containing 0.5 mg/L EGF or without EGF. Ten microliters of the cell suspension was seeded onto an amniotic membrane scaffold and incubated in a humidified incubator at 37 °C with 5% CO_2_ for 20 min. Subsequently, 0.6 mL of the culture medium was added to the receiving well of the plate, 0.1 mL of culture medium was added to the chamber, and the chamber was gently tilted into the receiving well. The culture medium was replaced the following day. Three wells were prepared for each culture condition. After 3–5 days of culturing, the samples were fixed with 3% glutaraldehyde and prepared for TEM observation. Briefly, samples were post-fixed with 1% osmium tetroxide, dehydrated through a graded acetone series (30%, 50%, 70%, 80%, 95%, and 100%), infiltrated with Epon812 resin, and embedded. Ultrathin sections (60–90 nm) were prepared, mounted on copper grids, and stained with uranyl acetate and lead citrate. Ultrastructural images were acquired using a JEM-1400FLASH transmission electron microscope (JEOL, Tokyo, Japan). Multiple representative fields of view were examined for each sample, and representative images showing cell–scaffold interactions were selected. Because the TEM analysis was performed for morphological characterization rather than quantitative evaluation, blinded analysis was not conducted.

### 2.10. Immunofluorescence Staining of Stem Cell Markers P63 and ABCG2

The preparation of the grafts and cell culture conditions were the same as those described in Section 2.7. Six wells were used per culture. After 5–7 days of culturing, when cells exhibited multilayer growth and signs of detachment, the culture process was terminated, and immunofluorescence staining was performed. Immunofluorescence staining was performed using specific antibodies against the limbal stem cell markers p63 and ABCG2. The primary antibody against p63 (rabbit anti-p63 antibody, Proteintech, Wuhan, China) was diluted 1:500 in PBST, whereas the primary antibody against ABCG2 (mouse anti-ABCG2 antibody, Invitrogen, Waltham, MA, USA) was diluted 1:1000 in PBST. After overnight incubation with primary antibodies at 4 °C, samples were washed with PBST and incubated with fluorescent secondary antibodies in the dark for 1 h. Alexa Fluor 488-conjugated anti-rabbit IgG secondary antibody (1:500) was used for p63 detection, and Alexa Fluor 555-conjugated anti-mouse IgG secondary antibody (1:500) was used for ABCG2 detection. Nuclei were counterstained with DAPI.

### 2.11. Statistical Analysis

All quantitative experiments were performed using independent biological replicates. Technical replicates within each independent experiment were averaged before statistical analysis and were not treated as independent samples. Data are presented as mean ± standard deviation (mean ± SD). Statistical analyses were performed using GraphPad Prism 9.0 software. For comparisons involving multiple groups and different treatment/time factors, two-way analysis of variance (ANOVA) followed by Tukey’s multiple-comparison test was used. A *p* value < 0.05 was considered statistically significant.

## 3. Results

### 3.1. CCK-8 Assay of FLSC Proliferation Under Different EGF Concentrations

**Figure 3:**
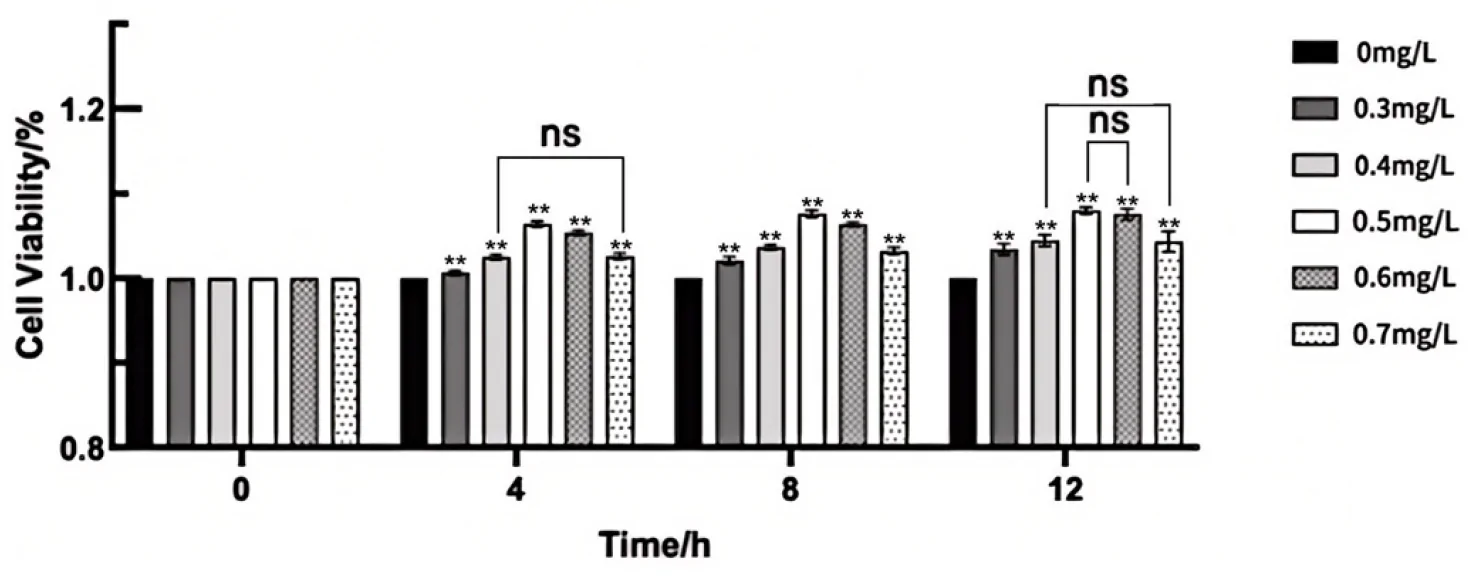
Proliferation of FLSCs under different EGF concentrations. Cell viability was measured at 0, 4, 8, and 12 h after treatment with different concentrations of EGF (0, 0.3, 0.4, 0.5, 0.6, and 0.7 mg/L). Data are presented as mean ± SD from three independent experiments, with ten technical replicates per group in each experiment. Statistical analysis was performed using two-way ANOVA followed by Tukey’s multiple-comparison test. ** *p* < 0.01; ns, not significant.

The viability of FLSCs treated with different concentrations of EGF was significantly higher than that in the EGF (0 mg/L) control group at 4, 8, and 12 h (*p* < 0.01) (Figure 3). Among all groups, FLSCs treated with 0.5 mg/L EGF exhibited the highest cell viability and presented significant differences compared with those in the other concentration groups (*p* < 0.01). Therefore, 0.5 mg/L EGF was selected as the optimal concentration and was added to the complete DMEM for subsequent experiments.

### 3.2. Preparation of the Growth Curve of FLSCs Under 0.5 mg/L EGF Treatment

**Figure 4:**
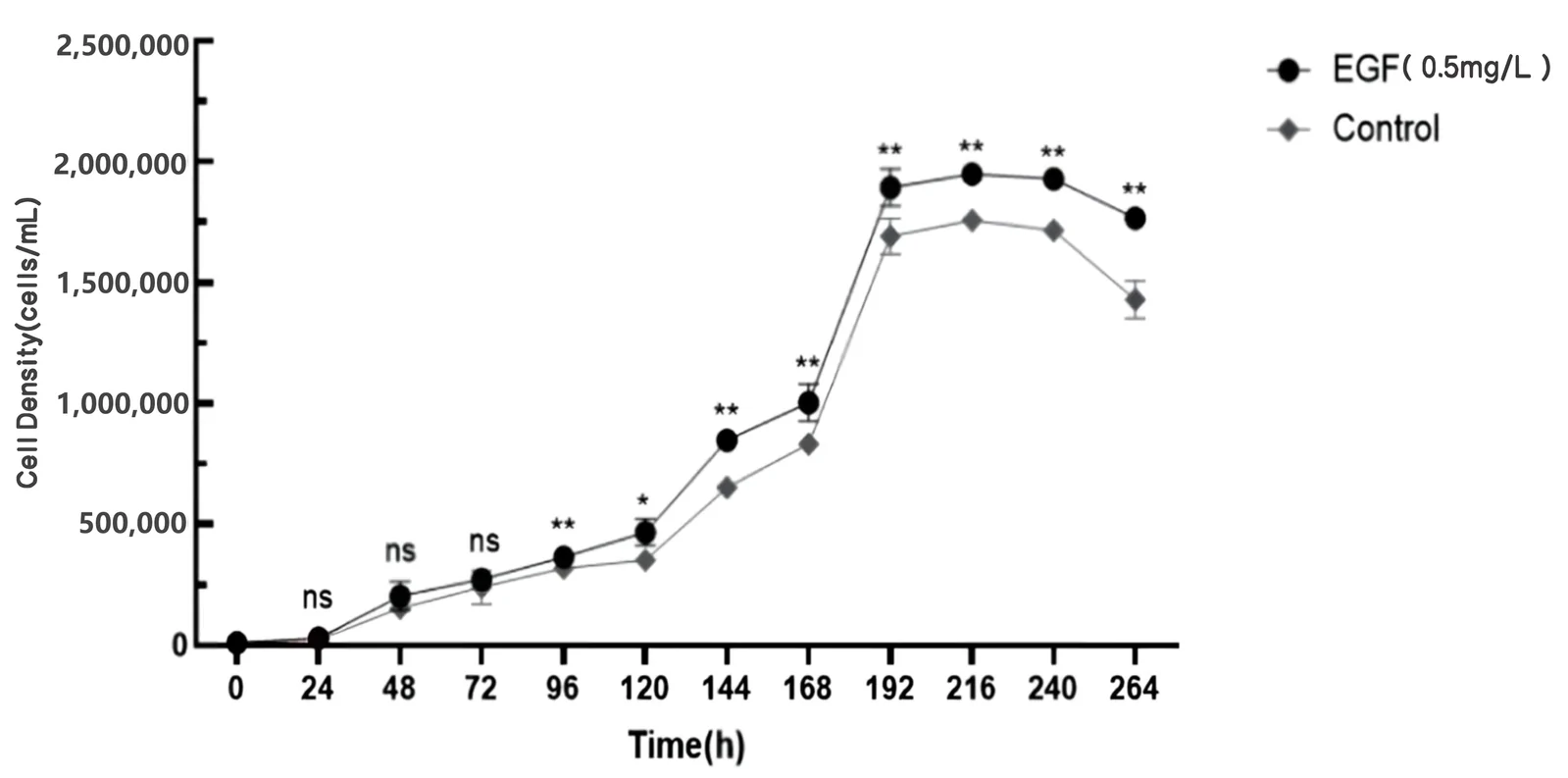
Growth curves of FLSCs treated with EGF (0.5 mg/L) or blank control. Cell numbers were measured at indicated time points. Data are presented as mean ± SD and were analyzed using two-way ANOVA followed by Tukey’s multiple-comparison test. *n* = 4. * *p* < 0.05, ** *p* < 0.01; ns, not significant.

The growth and proliferation of FLSCs in the EGF (0.5 mg/L) and EGF (0 mg/L) control groups are shown in Figure 4. The growth curve of the FLSCs exhibited a typical S-shaped pattern. The first 120 h corresponded to the lag phase, 120–192 h to the logarithmic growth phase, 192–240 h to the plateau phase, and 240 h onward to the decline phase. The population doubling time was calculated as TD = tlg2/(lg Nt − lg No), where No represents the initial number of seeded cells, and Nt represents the number of cells after t hours of growth. Based on this equation, the population doubling time of the FLSCs in both groups was calculated to be approximately 25.2 h.

### 3.3. Scratch Assay for Evaluating the Growth and Wound Closure of FLSCs

**Figure 5:**
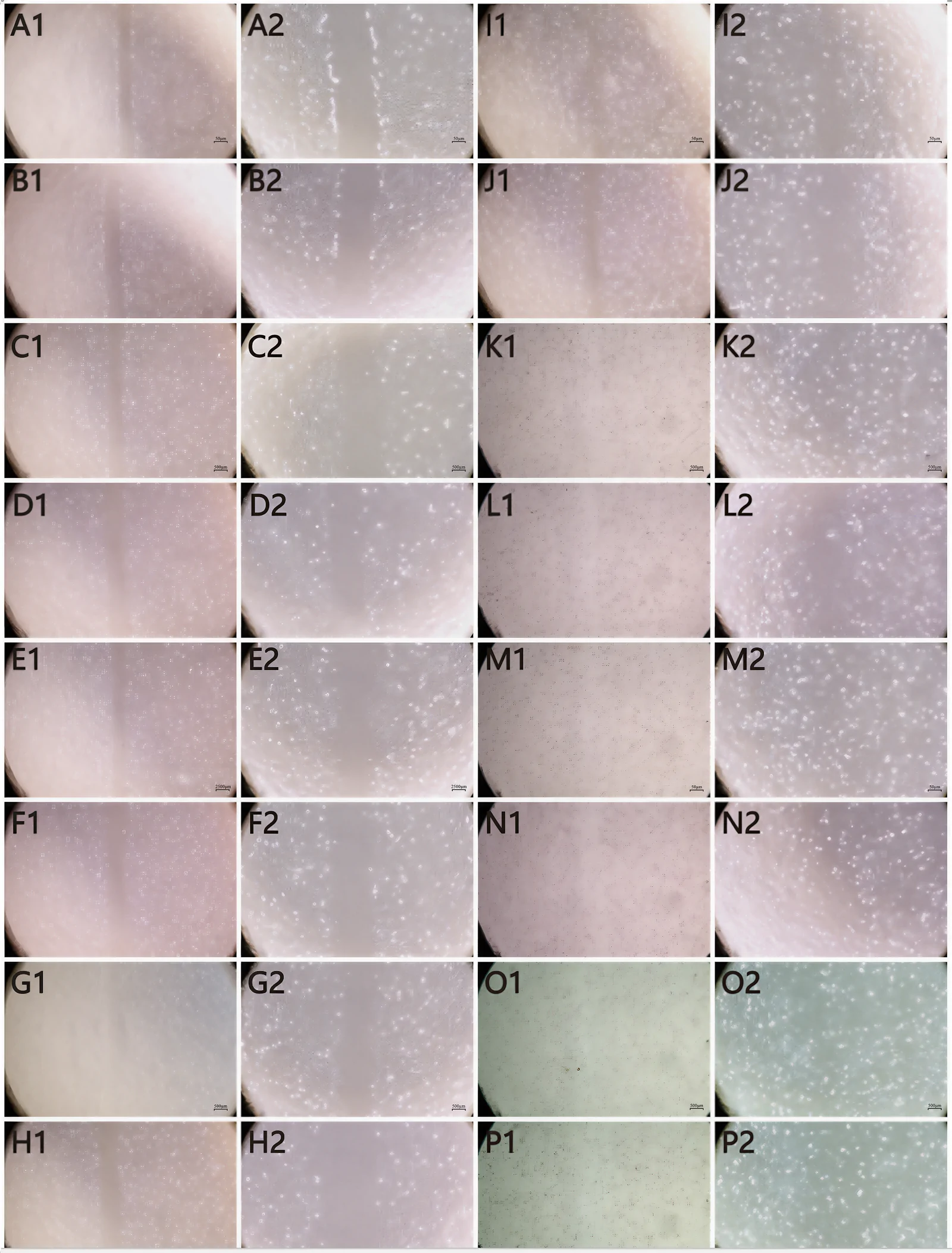
Wound-healing (scratch) assay for FLSCs with 0.5 mg/L EGF vs. control. Panels (**A1**,**A2**,**C1**,**C2**,**E1**,**E2**,**G1**,**G2**,**I1**,**I2**,**K1**,**K2**,**M1**,**M2**,**O1**,**O2**) show FLSCs cultured with 0.5 mg/L EGF at 0, 4, 8, 12, 24, 36, 48, and 60 h, respectively. Panels (**B1**,**B2**,**D1**,**D2**,**F1**,**F2**,**H1**,**H2**,**J1**,**J2**,**L1**,**L2**,**N1**,**N2**,**P1**,**P2**) show the blank control group at the corresponding time points, respectively. Images were acquired at 40× magnification with a scale bar of 100 μm and 100× magnification with a scale bar of 500 μm.

In the EGF (0.5 mg/L) group, FLSCs began to grow toward the scratch area in a small serrated pattern at 4 h. At 24 h, the scratch gap was narrower than that in the blank control group and had almost closed. Complete closure of scratches was observed at 36 h. In contrast, in the blank control group without EGF, gradual wound closure was observed from 8 h, and apparent gaps remained at 36 h. The scratch gap did not completely close until 60 h (Figure 5). Overall, scratch closure in the EGF (0.5 mg/L) group occurred 24 h earlier than that in the blank control group.

### 3.4. Light Microscopy Observation of FLSC Proliferation on a Lyophilized Feline Amniotic Membrane Scaffold

**Figure 6:**
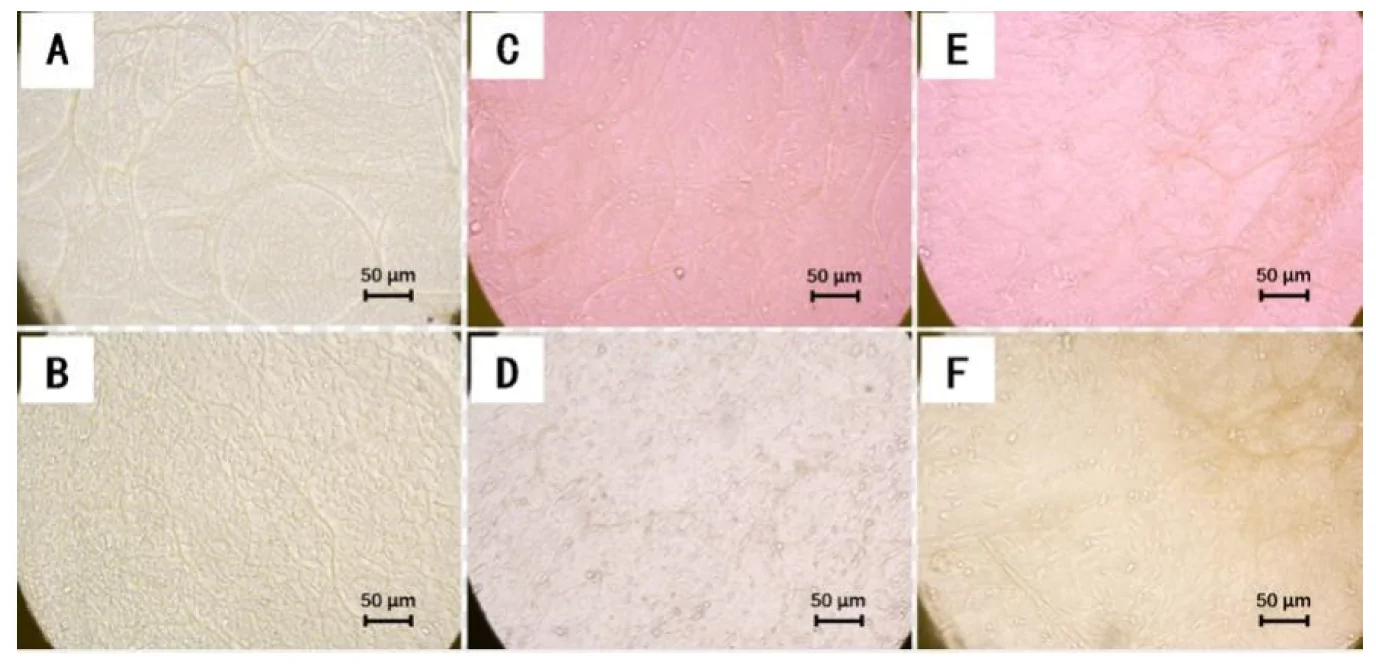
Light microscopy of FLSCs on a lyophilized feline amniotic membrane scaffold (EGF vs. control). Panel (**A**) shows the rehydrated amniotic membrane scaffold, panel (**B**) shows FLSCs initially seeded onto the scaffold, panels (**C**,**D**) show FLSCs cultured on the scaffold for 3 and 7 days in the EGF (0.5 mg/L) group, and panels (**E**,**F**) show FLSCs cultured on the scaffold for 3 and 7 days in the blank control group. Images were acquired at 200× magnification; scale bar = 50 μm.

Light microscopy revealed that the amniotic membrane scaffold was mainly composed of collagen fibers and that the epithelial surface exhibited an uneven mesh-like structure (Figure 6A). The FLSCs seeded onto the amniotic membrane scaffold initially appeared to be transparent, plump, and round (Figure 6B). On day 3 of culture, cells in both groups exhibited spindle-shaped morphology and had spread well on the scaffold. The number of adherent cells in both groups was lower than the initial seeding density. However, the cell distribution in the EGF group (Figure 6C) was notably denser than that in the blank control group (Figure 6E). On day 7, both groups exhibited evident proliferation compared with that on day 3. The cell distribution in the EGF group (Figure 6D) showed a visually higher cell distribution density compared with the blank control group (Figure 6F), with cells completely covering the amniotic membrane scaffold and exhibiting multilayer growth, whereas the blank control group covered approximately 80–90% of the scaffold surface.

### 3.5. Histological Observation of FLSCs Cultured on a Lyophilized Feline Amniotic Membrane Scaffold

**Figure 7:**
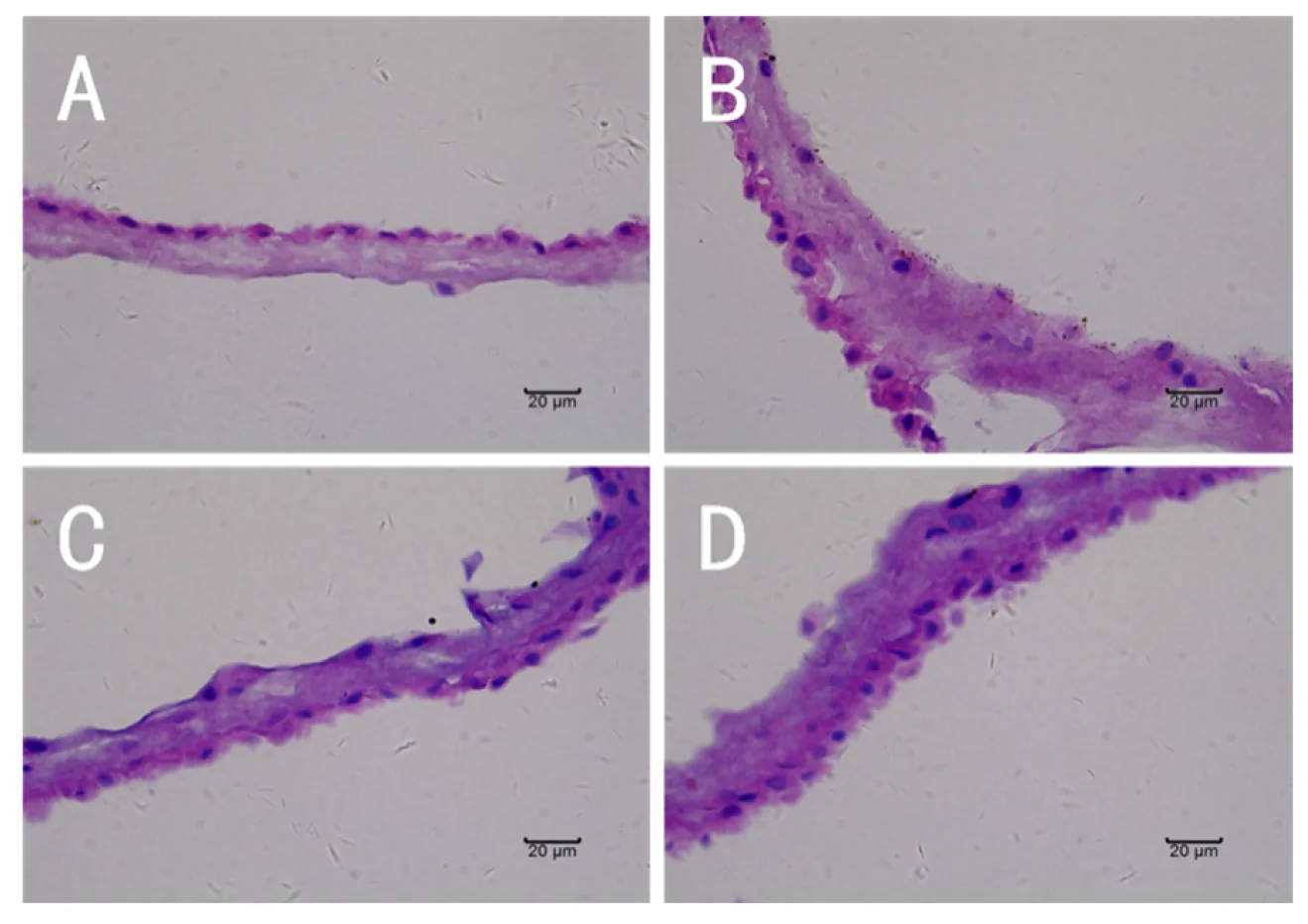
Histological examination of FLSCs seeded on the amniotic membrane. Panels (**A**,**B**) show FLSCs cultured on the amniotic membrane for 3 and 7 d in the blank control group, respectively, and panels (**C**,**D**) show FLSCs cultured on the amniotic membrane for 3 and 7 d in the EGF-treated group (0.5 mg/L), respectively. Images were acquired at 200× magnification; scale bar = 20 μm.

The histological results showed that the FLSCs cultured in both media adhered to the amniotic membrane scaffold and grew on day 3 after seeding. Most cells were embedded in the scaffold and arranged regularly. The number of cells in the EGF group (Figure 7C) was slightly higher than that in the control group (Figure 7A). On day 7, the number of cells in the EGF group increased substantially, exhibiting multilayer growth and neat arrangement (Figure 7D), whereas that in the blank control group increased only slightly and exhibited single-layer growth (Figure 7B).

### 3.6. Scanning Electron Microscopy Observation of FLSC Growth and Proliferation on a Lyophilized Feline Amniotic Membrane Scaffold

**Figure 8:**
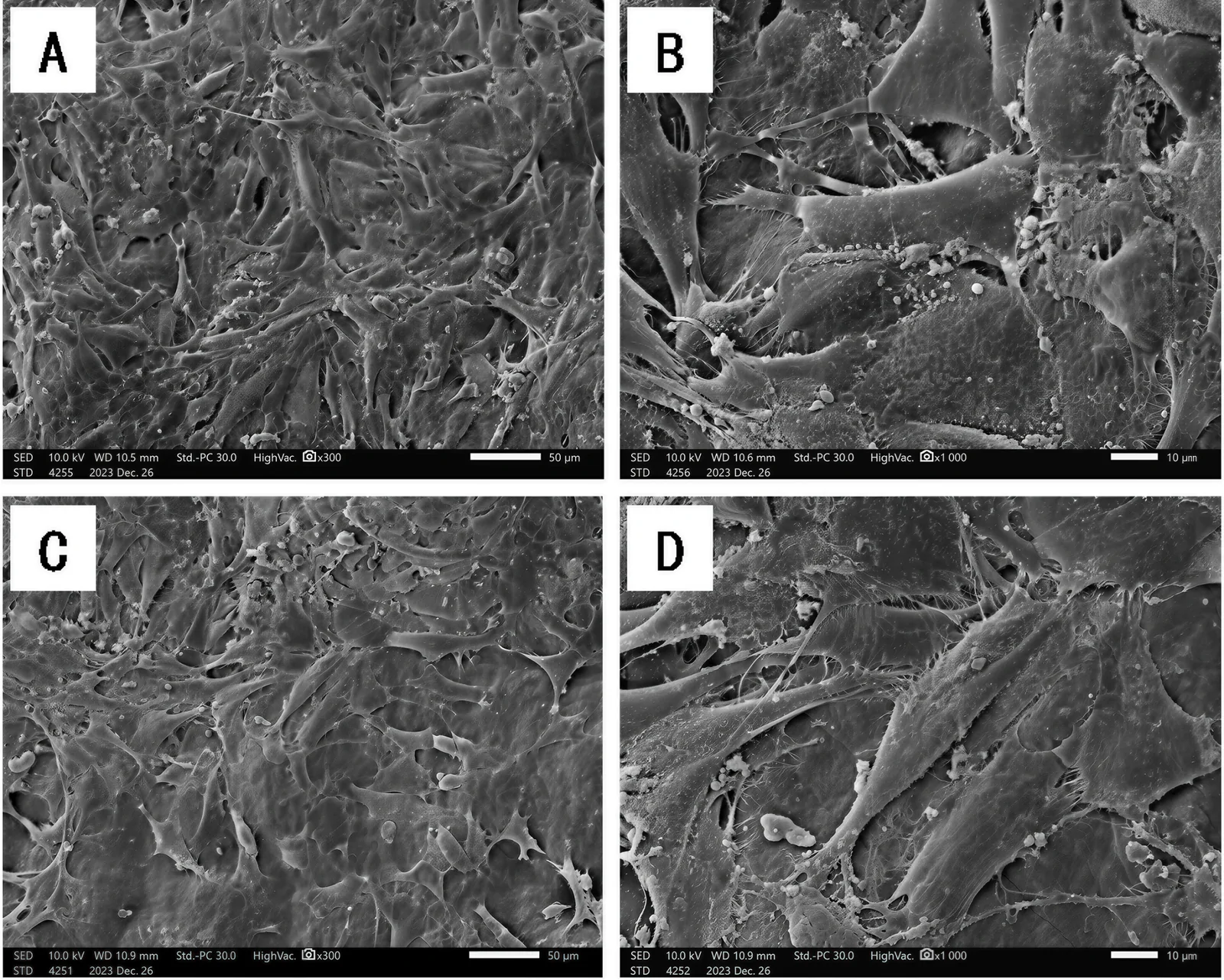
Scanning electron microscopy observation of FLSCs cultured on a lyophilized feline amniotic membrane scaffold. Panels (**A**,**B**) show FLSCs cultured on the scaffold for 7 days in the EGF (0.5 mg/L) group, and panels (**C**,**D**) show FLSCs cultured on the scaffold for 7 days in the blank control group. Images were acquired at 300× magnification with a scale bar of 50 μm and at 1000× magnification with a scale bar of 10 μm.

Under scanning electron microscopy, the FLSCs cultured on the amniotic membrane scaffold for 7 days were observed for both culture conditions. Cells exhibited spindle-shaped morphology and protruded from the scaffold’s surface. Filament-like pseudopodia extended from the cell surfaces, forming connections with adjacent cells and the amniotic membrane matrix. The cell bodies were flat and plump with clear and intact structures, good morphology, and no signs of rupture or deformation. The EGF group exhibited an apparently higher cellular distribution on the scaffold surface compared with the blank control group (Figure 8). However, the present study did not perform quantitative analysis of scaffold coverage, and the estimation of cell-covered area was based on morphological observation.

### 3.7. Transmission Electron Microscopy Observation of FLSC Growth and Proliferation on a Lyophilized Feline Amniotic Membrane Scaffold

**Figure 9:**
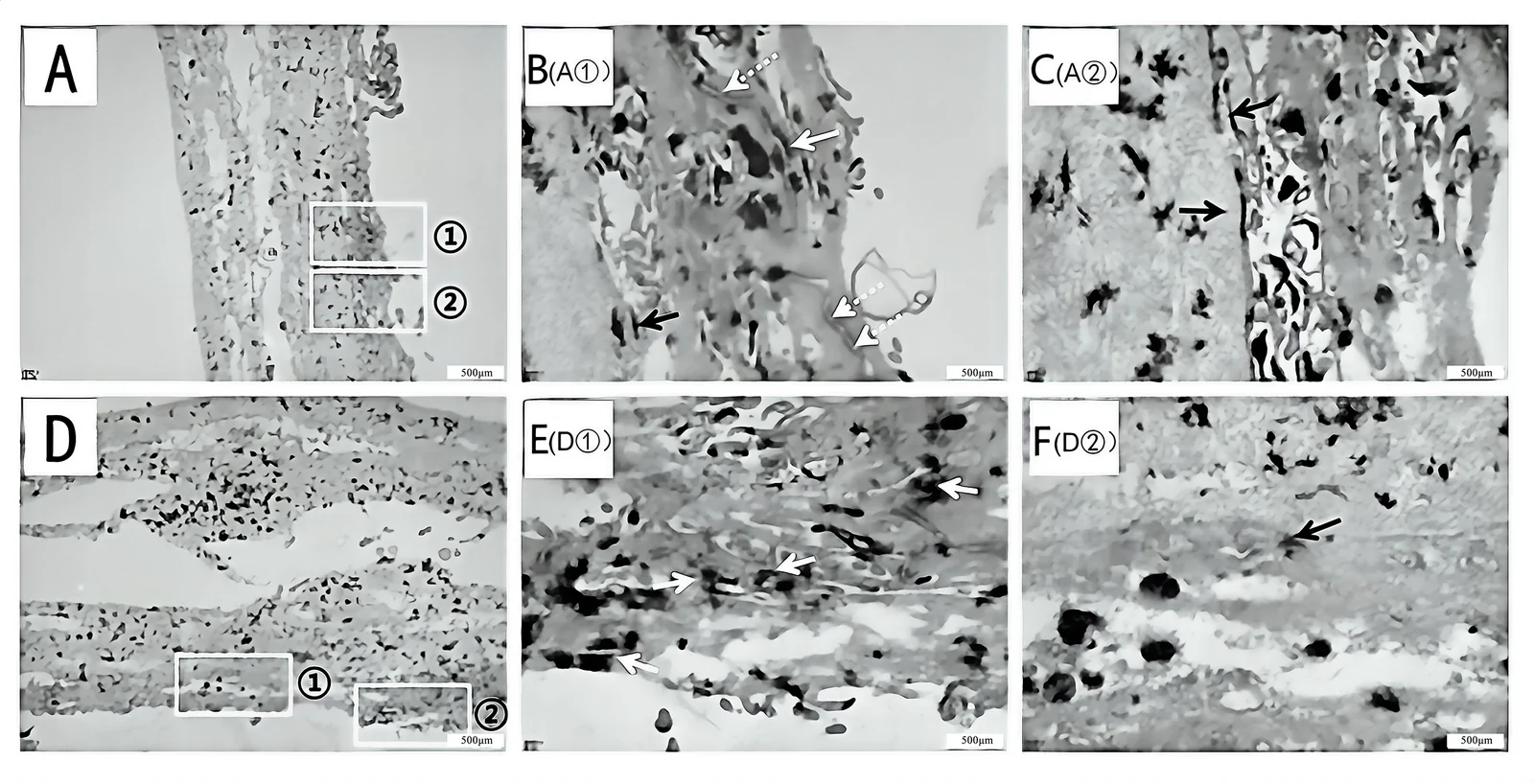
Transmission electron microscopy observation of FLSCs cultured on a lyophilized feline amniotic membrane scaffold. White solid arrows indicate bridging structures, black arrows indicate hemidesmosome-like structures, and white dashed arrows indicate gap junctions. Panels (**A**–**C**) show FLSCs cultured on the scaffold for 7 days in the EGF (0.5 mg/L) group, and panels (**D**–**F**) show FLSCs cultured on the scaffold for 7 days in the blank control group. Images were acquired at 4000× magnification with a scale bar of 2 μm and at 40,000× magnification with a scale bar of 500 nm.

FLSCs cultured on the amniotic membrane scaffold for 7 days were observed under both culture conditions via transmission electron microscopy. Cells grew on the scaffold and were not merely attached to its surface but established intercellular connections. As shown in Figure 9, bridging structures (white solid arrows) were formed between cells in both groups, with a higher density observed in the blank control group. In addition, hemidesmosome-like connections (black arrows) were established between cells and the amniotic membrane matrix in both groups, and gap junction-like structures (white dashed arrows) were observed in the EGF group.

### 3.8. Immunofluorescence Detection of FLSCs Cultured on a Lyophilized Feline Amniotic Membrane Scaffold

**Figure 10:**
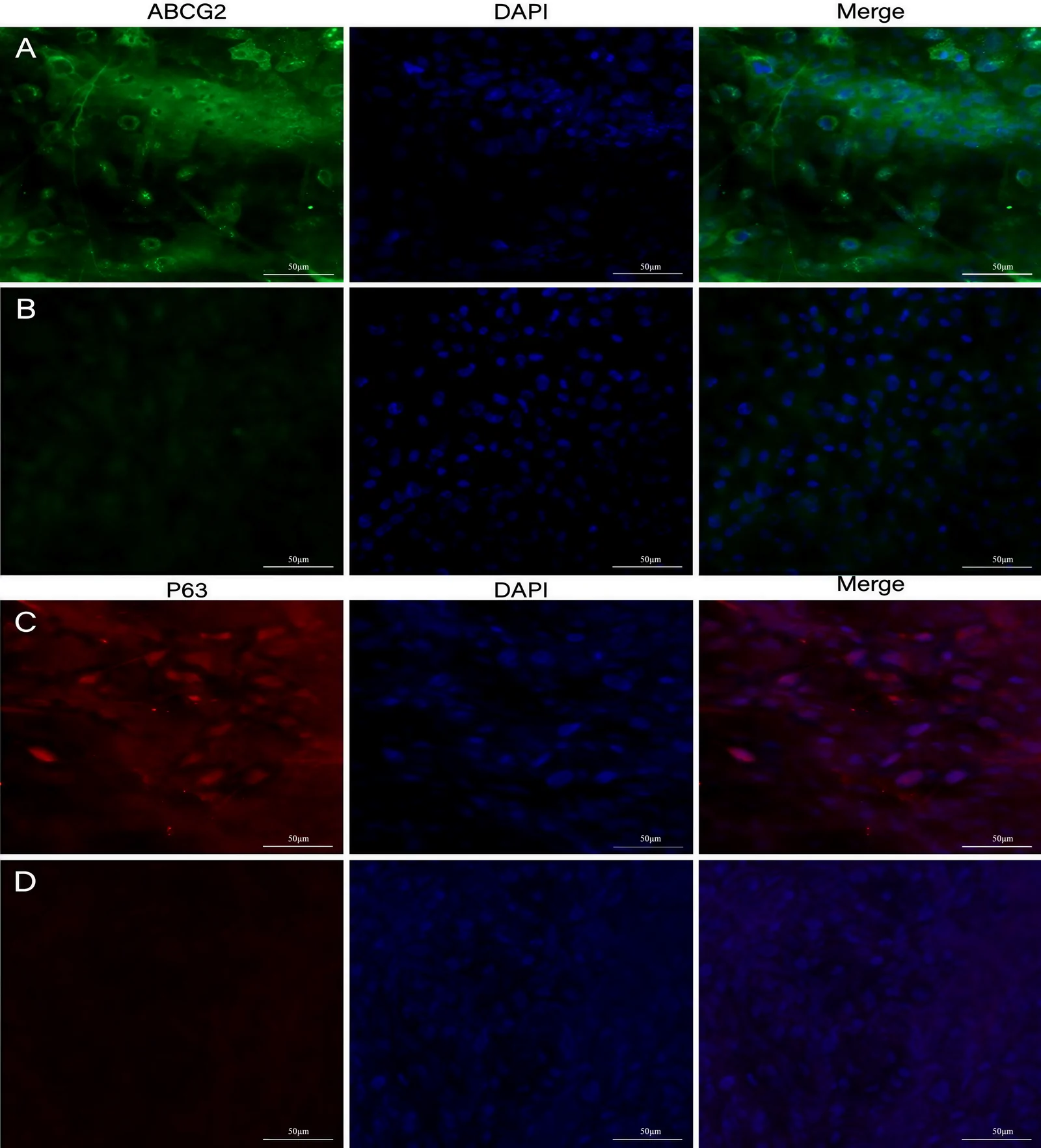
Immunofluorescence of FLSCs on the lyophilized feline amniotic membrane scaffold. Blue indicates DAPI nuclear staining, green indicates positive ABCG2 expression, and red indicates positive p63 expression. Panels (**A**,**C**) show immunofluorescence staining of FLSCs cultured on a lyophilized feline amniotic membrane scaffold, whereas panels (**B**,**D**) show immunofluorescence staining of the amniotic membrane scaffold alone. Images were acquired at 200× magnification with a scale bar of 50 μm.

The immunofluorescence staining results are shown in Figure 10. After the FLSCs were seeded and cultured on the amniotic membrane scaffold, ABCG2 (green) and p63 (red) exhibited strong positive expression. ABCG2 was mainly localized in the cell membrane and the cytoplasm, whereas p63 was mainly localized in the nucleus. The amniotic membrane scaffold without FLSC seeding displayed no red or green fluorescence, and only DAPI (blue) staining was observed. DAPI staining indicated that the FLSCs contained polygonal nuclei.

## 4. Discussion

Amniotic membranes are extensively used in ophthalmology, dermatology, neurosurgery, and other fields for repairing damaged tissues in both humans and animals because of their ability to inhibit inflammatory responses, reduce fibrosis, and promote healing [16,17,18]. The amniotic membrane is abundant, easily obtainable, and highly plastic in nature. In addition, the use of placental tissue that is discarded following pregnancy mitigates ethical and welfare concerns. However, preserving the integrity of fresh amniotic membranes presents considerable challenges. Preparing fresh amniotic membranes according to practical requirements is time-consuming and may increase the risk of microbial contamination. Fresh or cryopreserved amniotic membranes generally require continuous low-temperature storage conditions, which may increase transportation and storage complexity and limit their accessibility for clinical applications. In contrast, lyophilization can reduce water content, improve long-term storage stability, and facilitate convenient transportation and application without the need for continuous cryogenic conditions. Therefore, lyophilization has been increasingly explored as an alternative preservation strategy for amniotic membrane-based tissue engineering materials [19]. Compared with cryopreserved or glycerol-preserved amniotic membranes, lyophilized amniotic membranes offer several practical advantages for storage and clinical application. Cryopreserved amniotic membranes generally require continuous low-temperature storage and an uninterrupted cold chain, whereas glycerol-preserved membranes also require controlled storage conditions and additional processing before clinical use [20]. In contrast, lyophilization substantially reduces tissue water content and may improve storage stability, transportation convenience, and accessibility, making it potentially more suitable for the development of an off-the-shelf biological scaffold [21]. Previous studies have suggested that appropriate lyophilization procedures can preserve the extracellular matrix architecture and retain at least part of the biological activity of amniotic membranes, indicating that the structural integrity and cell-supporting capacity may be maintained during subsequent storage [22]. However, the stability of these properties is highly dependent on the lyophilization process, packaging conditions, storage temperature, storage duration, and rehydration procedure. The present study evaluated a pre-manufactured lyophilized feline amniotic membrane after rehydration but did not perform a systematic long-term storage study or directly compare fresh, cryopreserved, glycerol-preserved, and lyophilized membranes. Therefore, although the observed cell adhesion and proliferation indicate that the tested lyophilized scaffold retained sufficient biological activity to support FLSC growth, the long-term stability of its structural integrity and cell-supporting capacity cannot be conclusively established from the present data. Future studies should evaluate these properties after different storage periods and under standardized storage conditions and should directly compare lyophilized membranes with commonly used cryopreserved or glycerol-preserved amniotic membranes. Previous studies have suggested that appropriately lyophilized amniotic membranes can retain substantial portions of their extracellular matrix structure and certain biological activities, supporting their potential use in tissue engineering and ocular surface reconstruction applications [17,23,24,25]. Furthermore, the present study did not evaluate the preservation of endogenous growth factors within the lyophilized amniotic membrane, such as EGF, HGF, and TGF-β, compared with fresh amniotic membrane. Although previous studies have suggested that lyophilization may preserve certain bioactive components of amniotic membranes, quantitative evaluation of residual growth factor activity was beyond the scope of the present exploratory study. Future studies will further investigate the changes in bioactive factors before and after lyophilization to better characterize the biological properties of the scaffold. As the lyophilized feline amniotic membrane scaffold is a biological product derived from individual donors, potential donor-related and batch-to-batch variations should be considered. In the present study, only one representative batch of scaffold was evaluated, and reproducibility among independent membrane preparations was not systematically investigated. Future studies using multiple independent batches will be necessary to evaluate batch consistency and establish standardized quality control criteria for clinical translation.

In this study, a pre-prepared lyophilized biological amniotic membrane was observed under a light microscope. The membrane was composed of crisscrossed and regularly arranged fibers with a mesh-like surface containing abundant pores, a structure that meets the requirements of tissue-engineered scaffolds with a porous architecture that facilitates cell infiltration, adhesion, and fixation [26]. Interestingly, the mesh-like microstructure observed on the surface of the lyophilized feline amniotic membrane showed certain morphological similarities to network-like structures described in other biological contexts. However, whether this structure shares any mechanistic similarity with vasculogenic mimicry remains unknown and requires further investigation using specific molecular and functional analyses. Simple amniotic tissue can be transplanted or applied to sites of corneal injury in order to achieve favorable corneal repair outcomes. However, in clinical practice, many cases of severe corneal trauma are associated with a notable loss of limbal stem cells (LSCs), which may damage the Palisades of the Vogt area and lead to stem cell regeneration disorders. Therefore, seeding stem cells onto the amniotic membrane for in vitro culture and constructing engineered corneal epithelial grafts have emerged as important research foci. Rabbit LSCs seeded onto human amniotic membranes obtained after cesarean section and subsequently transplanted for rabbit corneal repair exhibited complete epithelialization of the graft and favorable prognosis [27]. The lyophilized amniotic membrane used in this study exhibited reduced water content and low immunogenicity while preserving its original tissue structure, physiological characteristics, and biological activity.

The present study successfully constructed a feline-specific tissue-engineered scaffold by seeding FLSCs onto a lyophilized feline amniotic membrane. The results demonstrated that FLSCs adhered well to the scaffold, maintained stem cell characteristics, and exhibited active growth. Immunofluorescence staining showed clear expression of the limbal/stem cell-associated markers ABCG2 and p63 after culture on the scaffold, indicating that FLSCs retained the expression of these examined markers under the tested culture conditions. However, because quantitative comparisons of marker expression before and after scaffold seeding were not performed, the present findings do not provide sufficient evidence to conclusively demonstrate quantitative preservation of stemness. Furthermore, scanning electron microscopy revealed that FLSCs established close interactions with the amniotic membrane matrix, including desmosome-like junction structures and extracellular connections, suggesting the formation of stable cell–matrix interactions. These findings indicate that the engineered construct possesses essential characteristics required for a bioengineered corneal epithelial graft.

EGF positively regulates corneal epithelial cell proliferation and promotes corneal stromal repair. During this process, stromal neovascularization can also be decreased. Even trace amounts of EGF can strongly stimulate cell growth and proliferation, inhibit apoptosis, and exert its biological effects in a concentration-dependent manner [28]. In this study, the CCK-8 assay results showed that the activity of FLSCs initially increased in an EGF-concentration-dependent manner, peaking at 0.5 mg/L. However, with a further increase in EGF concentration, cell proliferation began to decrease. This phenomenon is consistent with the results of previous studies by Yujie [29] and Wei [30]. This may be explained as follows: Moderate concentrations of EGF promote cell growth, whereas high concentrations lead to excessive receptor activation, increased extracellular matrix synthesis, and decreased cell adhesion. When cell adhesion is impaired, apoptosis may occur because adhesion-dependent signaling is essential for maintaining normal cellular behavior. When adhesion is disrupted, signal transduction is hindered, resulting in cell cycle arrest and the subsequent inhibition of cell proliferation. In addition, the number of binding sites is limited; when receptor binding becomes saturated, further increases in EGF concentration may not enhance proliferative activity.

Regarding the molecular mechanisms underlying the biological effects of EGF, previous studies in other cell models have demonstrated that the binding of EGF to its receptor induces receptor phosphorylation, subsequently activating various classical downstream signaling pathways, such as PI3K-PKB/Akt [31], FAK-ERK [32,33], and MAPK-ERK [34,35]. These established mechanisms provide a potential theoretical framework for explaining the enhanced proliferation and wound closure process of FLSCs observed in this study. For example, the activation of the PI3K-Akt pathway can induce gene expression and promote cell proliferation by regulating the release of NF-κB, whereas the FAK–ERK pathway plays a key role in regulating cell movement and inhibiting apoptosis [31,32,33], which may contribute to the improved wound closure observed in our experiments. Furthermore, research has indicated that EGF activates p-ERK1/2 to accelerate the G1/S phase transition, thereby promoting cell growth [36]. Although we did not directly evaluate the phosphorylation levels of these specific signaling molecules, the phenotypic outcomes (e.g., CCK-8 and scratch assay data) suggest that EGF likely modulates the growth, development, and wound closure process of FLSCs through these potential pathways. Future studies could further validate these mechanisms by using specific pathway inhibitors, such as LY294002 for PI3K/Akt inhibition, U0126 for MEK/ERK pathway inhibition, or PF-573228 for FAK inhibition. In addition, establishing an EGF-deficient or EGF-knockdown FLSC model may provide further evidence regarding the specific role of EGF-mediated signaling in regulating FLSC biological behavior.

Compared with conventional LSC transplantation approaches, this study was not intended to replace existing established therapies but rather to preliminarily explore the feasibility of a lyophilized feline amniotic membrane as a scaffold for FLSC culture. Unlike previous studies that mainly employed human-, rabbit-, or canine-derived amniotic membranes, this study focused on the development of a feline-specific amniotic membrane scaffold. As cats are important companion animals and ocular surface diseases are relatively commonly encountered in veterinary practice, studies involving feline-specific ocular-surface-tissue-engineered materials remain limited. Tissue-engineered scaffold materials may provide a favorable microenvironment for LSC attachment and growth while potentially improving graft preservation and clinical accessibility [37]. The results of this study demonstrate that lyophilized feline amniotic membrane could support FLSC adhesion, growth, and the formation of desmosome-like structures, suggesting its potential application as a scaffold for feline ocular surface tissue engineering. In addition, freeze-drying may provide a foundation for the future development of tissue-engineered grafts that can be stored long-term and conveniently used in clinical settings. Considering the clinical demand for ocular surface reconstruction in companion animals such as cats, the development of species-specific tissue-engineered scaffolds may potentially be translationally relevant in veterinary ophthalmology.

This study primarily focused on histological and ultrastructural observations to preliminarily evaluate the feasibility of the lyophilized amniotic membrane as a carrier for FLSCs, with particular emphasis on cell adhesion and the formation of desmosome-like structures. As an exploratory study, quantitative analyses remain limited, and wound closure analysis, cell density measurements, statistical evaluation of ultrastructural junction numbers, and quantitative analysis of molecular marker expression were not performed. In particular, quantitative comparison of p63 and ABCG2 expression between FLSCs before and after scaffold seeding was not performed using fluorescence intensity analysis, RT-qPCR, or Western blotting. Therefore, although the immunofluorescence results demonstrated continued expression of these limbal/stem cell-associated markers after scaffold culture, the present study cannot conclusively determine whether the quantitative level of the stem cell-associated phenotype was preserved. Future studies should include quantitative molecular and imaging analyses to further evaluate changes in FLSC phenotype before and after scaffold culture. In addition, biological safety evaluations, including long-term cell viability assessment, terminal differentiation analysis, tumorigenic potential evaluation, inflammatory response analysis, and systematic microbiological safety testing, were not conducted. Furthermore, the mechanical properties and surgical handling characteristics of the FLSC-loaded scaffold, including post-culture thickness, suture strength, and intraoperative manipulability, require further investigation. Future studies will integrate quantitative image analysis, molecular biological analyses, functional assays, extended culture experiments, microbiological safety evaluations, and biomechanical characterization to comprehensively assess the biological behavior and clinical applicability of the engineered scaffold.

In addition, because the lyophilized amniotic membrane scaffold was obtained as a pre-manufactured biological material, detailed manufacturing parameters, batch-to-batch reproducibility, long-term storage stability, residual growth factor content, and comprehensive physicochemical characterization were not evaluated in this exploratory study. Future studies should further investigate these properties, including structural integrity, cell-supporting capacity, and biological activity after different storage periods, and should compare lyophilized membranes with cryopreserved and glycerol-preserved amniotic membranes to establish standardized quality control criteria for clinical translation. Importantly, the present study was limited to in vitro characterization of the FLSC-loaded lyophilized feline amniotic membrane scaffold. No ex vivo corneal organ culture assay or in vivo feline corneal defect healing model was performed. Therefore, the present findings cannot directly demonstrate corneal wound-healing efficacy, graft integration, epithelial regeneration, inflammatory responses, or the long-term biological behavior of the construct following transplantation. These limitations should be considered when interpreting the potential clinical relevance of the present findings. Future studies will first evaluate the construct using appropriate ex vivo corneal organ culture systems and in vivo feline corneal defect models, with particular emphasis on graft integration, corneal epithelial regeneration, inflammatory responses, tissue remodeling, and long-term safety. Following adequate preclinical validation, translational studies may further evaluate the feasibility of the FLSC-loaded lyophilized feline amniotic membrane scaffold in cats with appropriate naturally occurring corneal surface diseases.

Collectively, the experimental design of this study followed a stepwise strategy, beginning with optimization of FLSC biological activity, followed by scaffold construction and finally biological characterization. This approach allowed a preliminary evaluation of whether the lyophilized feline amniotic membrane could function as a supportive biomaterial for FLSC-based ocular surface tissue engineering.

## 5. Conclusions

In this study, a lyophilized feline amniotic membrane scaffold loaded with FLSCs was successfully constructed and evaluated in vitro. EGF significantly promoted FLSC proliferation and wound closure at a mass concentration of 0.5 mg/L and maintained stable growth characteristics. The FLSCs adhered well to the amniotic membrane scaffold, formed stable intercellular and cell–matrix junctions, and exhibited multilayer growth. Immunofluorescence staining confirmed strong expression of the stem cell markers ABCG2 and p63 after seeding on the scaffold. These findings suggest that the engineered construct possesses several preliminary characteristics desirable for a potential bioengineered corneal epithelial graft. Further validation of its efficacy will be pursued using appropriate animal models or clinical cases in subsequent studies. These studies will evaluate graft integration, corneal epithelial regeneration, inflammatory responses, tissue remodeling, and the long-term biological behavior of transplanted FLSCs to further determine the clinical applicability of the engineered scaffold.

## Data Availability

The original contributions presented in this study are included in the article. Further inquiries can be directed to the corresponding author.

## Abbreviations

The following abbreviations are used in this manuscript:

ABCG2	ATP-binding cassette subfamily G member 2
Akt	Protein kinase B
CCK-8	Cell Counting Kit-8
CO_2_	Carbon dioxide
DAPI	4′,6-diamidino-2-phenylindole
DMEM	Dulbecco’s modified Eagle’s medium
EGF	Epidermal growth factor
ERK	Extracellular signal-regulated kinase
FAK	Focal adhesion kinase
FLSCs	Feline limbal stem cells
LSCs	Limbal stem cells
MAPK	Mitogen-activated protein kinase
NF-κB	Nuclear factor kappa B
PBST	Phosphate-buffered saline with Tween-20
PI3K	Phosphoinositide 3-kinase
SEM	Scanning electron microscopy
TEM	Transmission electron microscopy
Wnt	Wingless-related integration site
YAP	Yes-associated protein
